# Wheel running predicts resilience to tumors in old mice

**DOI:** 10.1080/20010001.2019.1676104

**Published:** 2019-10-10

**Authors:** Lida Zhu, Juan Wang, Christina Pettan-Brewer, Warren Ladiges, Jorming Goh

**Affiliations:** a Department of Comparative Medicine, School of Medicine, University of Washington, Seattle, WA, USA; b Department of Physiology, Yong Loo Lin School of Medicine, National University of Singapore, Singapore; c Centre for Healthy Ageing, National University Health System, National University of Singapore, Singapore

**Keywords:** Aging, resilience, aging mouse model, wheel running, cancer

## Abstract

Aging intervention studies are hampered by the lack of predictive measures for determination of individuals at risk of age-associated chronic disease. Assessment of physical resilience could be informative in this regard, especially for age-related diseases such as cancer. Voluntary wheel running is a mildly stressful physical activity that is easily quantifiable in the mouse but has not been studied as a predictor of resistance to tumor invasiveness with increasing age. Male C57BL/6 mice in cohorts of 4, 12, 20, and 28 months of age were allowed access to a slanted in-cage running wheel for 3 days. Three months later, mice were injected subcutaneously with B16 melanoma tumor cells and followed for two weeks before harvesting. No relation was observed between running distance and tumor burden in the 4-month age group. The 12-month age group showed a trend, and the 20- and 28-month age groups showed a negative correlation (*P* < 0.05) between running distance and tumor burden. Mice in the 20-month age group that ran longer distances had lower tumor invasive scores compared to mice in the same age group that ran shorter distances. In conclusion, short term exercise capability could be a marker for resilience to cancer, and possibly other age-related disease conditions, in mice.

Healthy aging can be evaluated by measuring the functional status of various organs and physiological systems that are important in physiological performance, but this provides information only at old age. Physical resilience, defined as the ability of an organism to respond and quickly resolve physical stress, can be measured at younger ages [1] by various types of stressors, for example increased skeletal muscle activity during exercise. Voluntary wheel running by mice is a mildly stressful physical activity that is easily quantifiable [2,3]. We hypothesized that short term voluntary wheel running would align, in an age-related manner, with melanoma tumor behavior. C57BL/6 male mice 4, 12, 20 and 28 months of age were obtained from the National Institute on Aging contract facility (Charles River, Inc.), and group-housed under specific pathogen-free conditions.

The study was approved by the University of Washington Institutional Animal Care and Use Committee. After a one-week acclimation period, mice were transferred to individual cages containing a slanted running wheel (Med Associates Inc.) capable of wirelessly recording activity data with details on speed, distance and time spent running. Wheels were locked for two days to allow for acclimation, and then unlocked for three days to measure running activity. Mice were returned to their group cages and held for three months to decrease the possibility of any anti-tumor effect of wheel running [3]. Mice were then injected with 5 × 10^5^ B16F0 melanoma tumor cells (ATCC) subcutaneously into the left inguinal space and the right axillary space as previously described (Pettan Brewer et al., 2012). At 14 days post-injection, mice were sacrificed, and tumor burden was calculated as a percent of tumor mass to total body mass.

**Figure 1.**
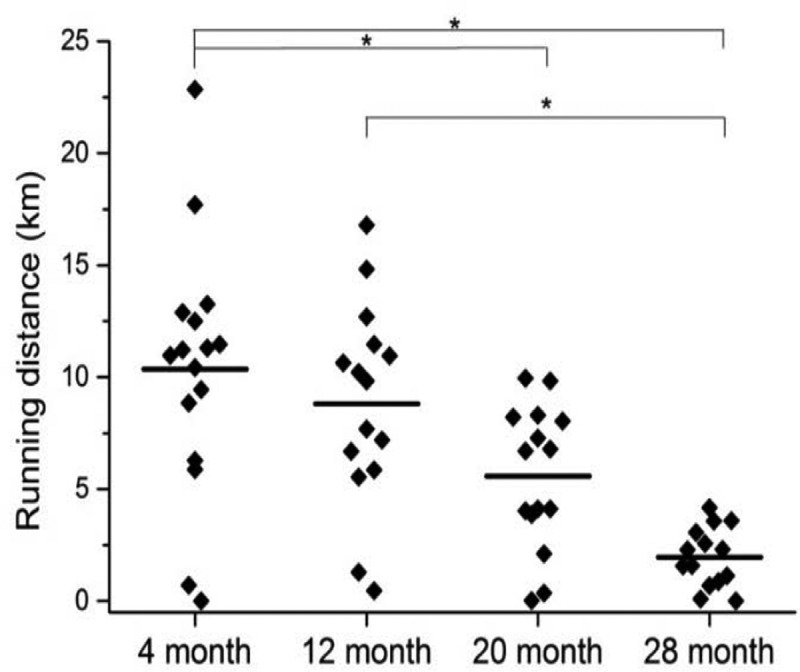
Running distance decreased with increasing age. Mice were allowed to access running wheels for three days. Mice at 16 months showed significant differences in running distances. Statistical analysis of data was performed by 1-way ANOVA and the significance of the difference was defined and considered significant for *P < 0.05.

By the end of the study, cohorts were 8, 16, 24, and 32 months of age with N = 12, 13, 13 and 6, mice per group respectively. Resected tumors were placed in 10% neutral buffered formalin, stained with hematoxylin and eosin, and then graded for invasiveness using a scoring range of 1 to 3, with 1 being least invasive and 3 being most invasive as previously reported (Pettan brewer et al., 2013). Slides were graded by scoring at least five microscopic fields per tumor from at least five mice per cohort.

The three-day wheel running distance decreased with increasing age (Figure 1). This result was expected because mobility generally declines with increasing age [4,5]. A significant difference (*P* < 0.05) in running distance was found between groups at 4 and 20 months, 4 and 28 months, and 12 and 28 months. All mice developed tumors after injection. Spearman’s/Pearson’s correlational analysis showed a negative correlation between distance ran and tumor growth in old mice but not young mice (Figure 2). The 4 and 12-month old mice showed no relation. However, a strong negative correlation was found in the 20-month group (*P < *0.01), while the 28-month group showed a similar correlation, yet only reached the *P* < 0.1 significance level, possibly due to the small number of mice left in that cohort. Seven mice in the 20-month age group that ran an average of 7.5 km over three days had an average tumor invasive grade of 1.3 ± 0.2 while six mice in the same age group that ran an average of 2.3 km had an average tumor invasive grade of 2.6 ± 0.3, *P* ≤ 0.05. As a representative example, a clear boundary between tumor and surrounding tissue with a visible fibrotic encapsulation can be seen in a mouse with long running distance in the 20-month group (Figure 3(a)). A mouse in the same age group that ran the shortest distance had evidence of invasiveness as suggested by penetration of the fibrous capsular layer (Figure 3(b)).

**Figure 2.**
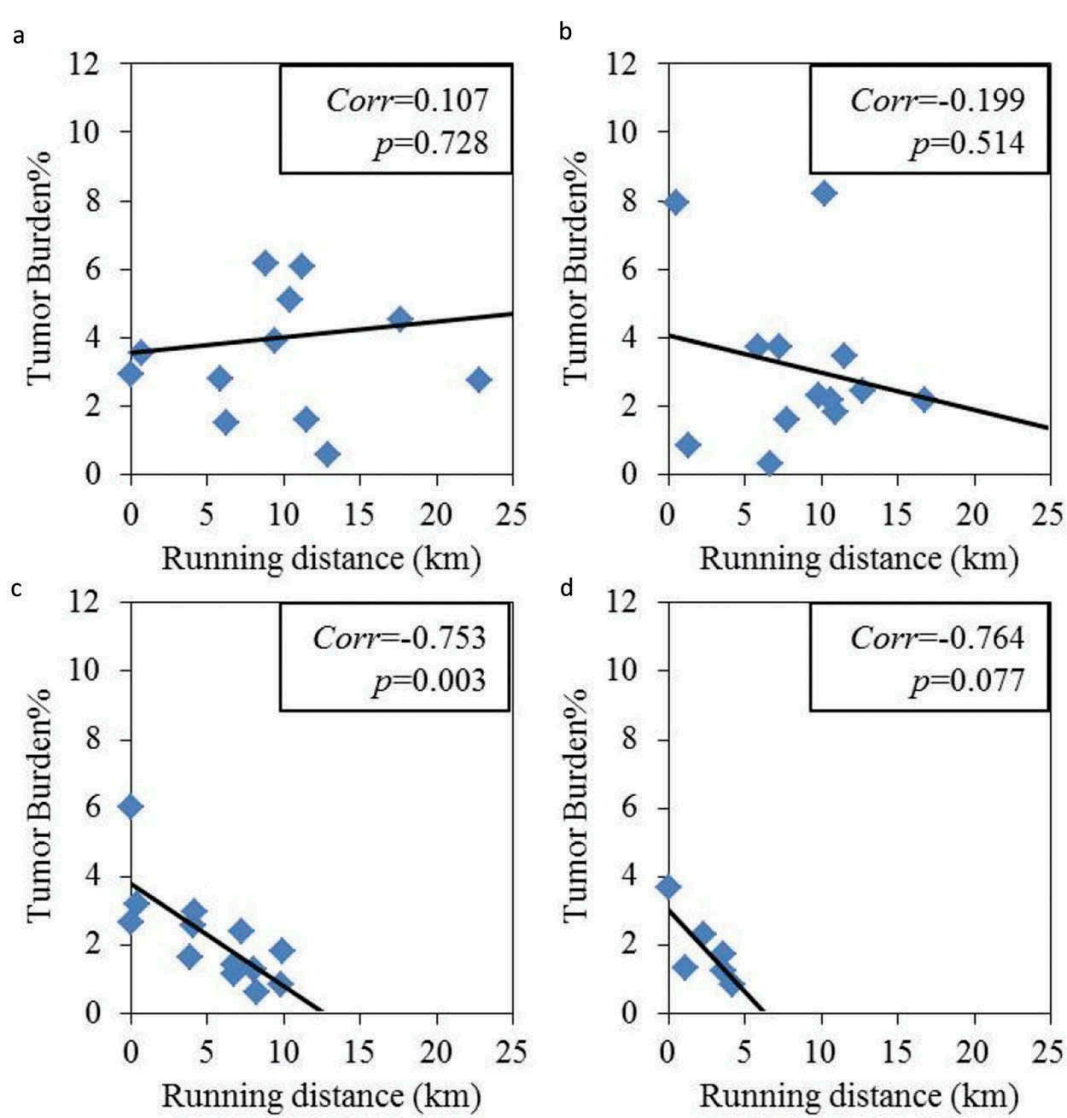
Three-day running distance correlated with decreased tumor burden at older ages. (a) The 4-month group showed no clear relation between distance ran and tumor burden. (b) The 12-month group showed a trend, while (c) the 20-month group and (d) the 28-month group showed a linearly decrease in tumor burden with increased distance ran. The line shows the linear fit. The correlation analysis of running distance vs. tumor burden was performed using JMP Pro version 14.

**Figure 3.**
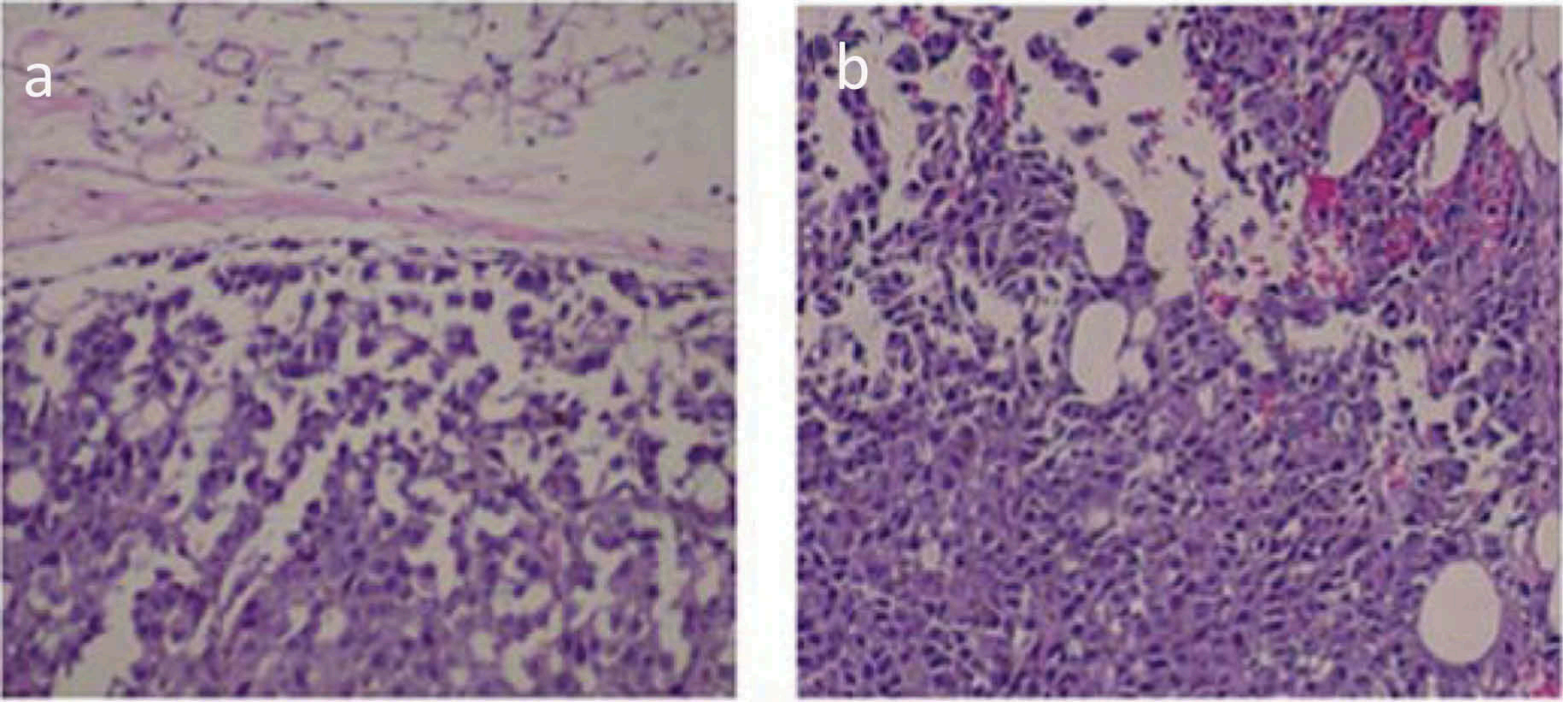
Melanoma tumors were less invasive in older mice that had high three-day running distances compared to mice with low three-day running distances. (a) Representative tumor from a high distance three-day runner in the 20-month age group shows a walled-off noninvasive tumor. (b) Representative tumor from a low distance three-day runner in the 20-month age group shows tumor cells invading space through and beyond the host-response barrier. Tissues were stained with hematoxylin and eosin and examined microscopically at 100 X magnification.

Compared to forced muscular tests such as treadmill or grip strength, voluntary wheel running measures both aerobic capacity and running motivation. It is more representative of physical ability, as it also reflects brain function and neuromuscular signaling [6]. Aging is accompanied by a progressive loss of physical ability, which can lead to frailty and weakness [7,8]. Frailty does not only result from the loss of muscle mass, but may also be caused by neurological dysfunction and changes in the intrinsic properties of muscle. For example, the loss of desmin reduces mouse running time, speed, and distance [9], yet desmin expression increases significantly with aging whereas muscle mass declines with aging [10]. The age-related decline in body strength includes multiple pathways such as inefficient neuro-signaling for muscle activation, muscle force transmission, and contractile protein decline [11]. Mice with greater running motivation should have higher daily physical activity, which could enhance immune function and therefore increase resilience with increasing age [12]. Interestingly, the correlation between running distance and tumor growth varied with age, and such variation was most obvious between 12 and 20 months. This age range suggests a critical period where the relation with resilience starts to appear, and may represent an opportune time for interventions to prevent further deleterious physical changes

In summary, running distance over 3 days correlated with resilience to tumor invasiveness in C57BL/6 male mice that are older than 12 months of age. This preliminary observation suggests that voluntary wheel running might serve as a useful functional test for studying resilience to age-related tumor behavior and possibly other age-related disease conditions in mice.
